# Southern Dental Association

**Published:** 1884-06

**Authors:** 


### SOUTHERN DENTAL ASSOCIATION.

SIXTEENTH ANNUAL MEETING, HELD AT LEXINGTON, KY., MAY 6, 7, 8 AND 9, 1884.

REPORTED EXPRESSLY FOR THE INDEPENDENT PRACTITIONER.

### FIRST DAY.

The Association met in the Y. M. C. A. rooms, Tuesday, May 6, there being present, beside representatives from the various Southern States, a number from Ohio and Indiana, and one from Illinois.

At two o'clock P. M. the convention was called to order by the President, Dr. McKellops, of St. Louis. Prayer was offered by Dr. J. Taft, of Cincinnati, and the President presented his address.

Dr. A. O. Rawls, of Lexington, chairman of the Executive Committe, then delivered an address of welcome.

In the absence of the Secretary of the Association, Dr. J. S. Walter was elected Secretary *pro tem.*; and the minutes of the last session being read, were approved.

The Executive Committee reported the following order of meeting:

Morning session from nine to twelve o'clock.

Viewing instruments and material on exhibition by various dealers, twelve to two o'clock.

Afternoon session, two to five o'clock.

Night session subject to order.

### DENTAL EDUCATION.

Prof. R. B. Winder, dean of the Baltimore College of Dental Surgery, read a paper on Dental Education.

He first considered what education is—its purposes and objects. Practically and theoretically it has been found necessary to have general, special, and ornamental education. Knowledge is confined to nature and her laws. As knowledge increases, the attaining of a general education becomes more difficult. The object being success in life, a young man should first be taught the essentials of success in his especial calling, and having attained these, it is his duty to cultivate and enlarge his mind in every way. Then he denounced in strong terms the wrongs perpetrated under the name of "female education," and argued that woman should be educated in the essentials of success in her pathway in life. Defining and explaining special and ornamental education severally, he held that that man is best educated practically who has most thoroughly mastered the essentials of success in his field of living—who is a success in his specialty. This brought him to dental education, and the founding of the Baltimore College of Dental Surgery by Chapin A. Harris, forty-six years ago, since which time many dental colleges have arisen, and many medical schools have added dental chairs to their faculties. This has caused great diversity of opinion as to the best method of educating dentists. He combated the idea that dentistry is a specialty of medicine. As did surgery, so has dentistry come to the front rank. Dentistry stands on these pillars—mechanics, artistic taste, and theory, named in order of their value. A dentist deficient in either will never be a success. Practical education is necessary, and everything unnecessary must be discarded in teaching dentistry. Diagnosis is most important. Graded courses should be established, and diplomas should be granted on knowledge, not on the time spent in pursuit of it. There is a necessity for the cultivation of artistic taste, involving a study of nature. There is need for a study of physiognomy (especially as most of the muscles of the face have their origin and insertion in the maxillary bones), for the acquiring of an accurate knowledge of the laws relating to physical beauty, and a skillfulness in carrying out their demands, if we would give dentistry an honorable, reputable and enviable place among the professions.

Drs. Taft and Smith, of Cincinnati, Dr. Patrick, of Belleville, Ills., and others spoke on the subject, which was left open for further discussion.

Dr. H. A. Smith, of Cincinnati, and Dr. J. S. Cassidy, of Covington, Ky., were elected active members, and Dr. John J. R. Patrick, of Belleville, Ills., an honorary member of the Association, which then adjourned to meet at the office of Dr. Rawls at eight o'clock.

EVENING SESSION.

After opening, Dr. C. E. Dunn, of Louisville, was elected a member.

Dr. Patrick was called on to speak on operative dentistry in relation to regulating teeth. He said there were so many questions connected with the regulating of teeth that it was difficult to speak definitely. No two cases are alike in the displacement and the condition of the surrounding parts. Besides, there are defects on all sides. The only certain guide is a correct knowledge of the parts surrounding the teeth to be regulated and shifted. That teeth can be changed in position there can be no doubt. Nature has set the example by shifting them out of place; art can use means to get them in their places. The question of how to do it remains with the individual, to a great extent. Whatever successful means are used, they must inevitably follow certain fixed laws of mechanics, known ever since Archimedes explained the operation of the lever. The rest is a question of judgment. Time is absolutely necessary. Every tooth has a cell within the alveolar process. A tooth of a single fang has only one cell, divided into two parts; a primary cell, embracing the neck of the tooth, and the fang cell. The formation is more clearly seen in teeth that have more than one cell, as the bicuspids and molars. As all the teeth of a set have a differently formed cell, of course, if you wish to shift teeth, they must be clearly understood. Take, for instance, the first bicuspid of the superior maxilla. It sometimes has two fangs, always two canals. When separate fangs are wanting they are united. The second bicuspid very seldom has its fangs separated. The first superior molar has two fangs, the first external, the other internal; a primary cell in the neck of the tooth, and a dental septum that separates it from the adjoining tooth. The canal passes up through the tooth. There it has a fang septum running up into the bifurcation. In shifting that tooth you disturb every one of those cells and septums. The same tooth in the lower jaw has two fangs cognate, four separate canals, sometimes bifurcated, sometimes with four fangs. As a rule, there are two fangs cognate, posterior and anterior. As all teeth have different surroundings, and are different as to support, thickness, form, and size, they require to be differently treated, to be dealt with intelligently. Once having begun, keep at it. You will have to trust in Providence. You might as well go slow. As one side excavates, the other will deposit a callous, which will become bone. When you change the position of the cell you change the position of the tooth; for wherever the tooth goes, there the cell accompanies it. You will find that with anomalous teeth there are anomalous cells. You cannot always tell by the crown the condition of the fangs. When the crown is very large the fangs may be very small; and when the crown is very small the fangs may be very large. The teeth of the human mouth you will very seldom find equally balanced, but showing as much difference as between the appearance of two separate individuals. In shifting teeth you must have one fixed point (two are better), and one movable point. You can move teeth between two fixed points in any given direction, whether you use the split plate or any other kind of method. The plate generally has more than one fixed point—it should never have more than two. A molar tooth is just as easily moved as a bicuspid. When you have two fixed points, one on either side, the probabilities of success are better.

There is a difference between the superior and inferior human maxilla. The outer wall of the superior maxilla is the weaker. In a large majority of cases there are signs of the fangs of the front teeth, especially the canines, protruding on this wall externally. It is not so in the lower jaw. In the upper jaw the outer side is the weaker; the inside the stronger, having a thicker wall, reinforced by a blade process which comes up to the alveolar process. The teeth are easier to move outward than inward. In the lower jaw there is also a line of weakness and a line of strength. The molars are reinforced by a hard, bony wall, termed the oblique ridge, and are thrown inward. In extracting, note that they should be thrown inward, as it is almost impossible to force them outward, on account of that ridge. The second bicuspid takes just a corner of it—the internal anterior corner. The process is thinner on the inside till you come to the second bicuspid, or right to the weakest point; then the inner process of that same ridge is the thickest. The anterior is thickest inside, and the posterior thinnest. It forms a figure 8. That is the reason the maxillaries press more at that place. If you must move a tooth there, you will have to go slow, because of the great length of the fangs, and the proximity to the surface. If much force is used, inflammation will be induced, which it may take months to control. The six front teeth are fortunately those we have most to work upon, and we have the others to anchor to.

Dr. Patrick answered a number of questions in regard to the extraction and movement of teeth. He wondered that more injuries are not done in pulling teeth, because so many muscles attach to the jaws. In making a plate for the lower jaw a very nice impression is needed, and not too low, lest it cut the muscles.

Leave young teeth alone. Give them time, even when you do regulate them. When you disturb a tooth, hold it. Never use rubber except to get the tooth in place; then something firmer to keep it there. He would not attempt to regulate permanent teeth under fifteen years. Deciduous teeth have to be taken between the ages of four and five. Before four the roots are not formed, and after five they are beginning to absorb. The wearing away of the little cusps on the incisors is a good indication that the fangs have calcified, and the teeth should never be removed till this is the case. The same rule will help in observing as to the time when the molars have developed. You have a sure sign in the incisors. He would take a patient at twenty-five, or thirty, or forty—if healthy.

If the tooth will not develop, make room for it to develop. A buried incisor should be relieved, even if the cusps are not visible. There are exceptions to every rule.

To fasten the ends of this wire (Dr. Patrick's apparatus), first select a back tooth, then make a piece of gold plate to fit snugly, and attach the slide to it with the screw. It does not matter if it doesn't fit exactly tight, provided it embraces the tooth somewhat. When you bring the strain between these bands, and the spring relaxes, it may drop out as the tooth moves. Then screw it up. As the bicuspids are out of line, band this inside, and it presses them in.

If an upper tooth pass between two lower teeth, bring these two lower ones together. Get that upper tooth loose and they won't come together. Put a little cross-bar to keep it there. His machine is only to move the tooth, not to keep it there.

He did not propose to do anything impossible, but might move the lower molars out with this machine, if he kept it on long enough.

As to hereditary development, he asked to be shown a crooked jaw and he will show crooked teeth. If you have a gothic jaw you have irregular teeth. Show him a placenta ill-formed, and he will show a monstrosity. It cannot be otherwise. So with the teeth. The same law governs the teeth that governs the hair of your head. You must work inside that law of government. In regard to heredity, we find irregularity of the teeth in all races of men. The lower you go the more regular the teeth are. The Australian has more regular teeth than the Congo negro. His teeth resemble those of the chimpanzee and gorilla, both as to the form of the jaw and the teeth, more than do those of the European; they have less fissures. The molars have very few fissures; indeed, only indications. The enamel is strong, and the teeth do not decay. The upper jaw rather approaches toward the ourang's. The higher we come the more mixed the races become. In America, Africa and Asia you find innumerable and diverse races. The different races must have crossed, and, in crossing, got imperfections as well as perfections. The European races are mixed probably more than any of the others.

(TO BE CONTINUED.)

